# Tetra­ethyl­ammonium bromidotricarbon­yl(tropolonato)rhenate(I)

**DOI:** 10.1107/S1600536810024505

**Published:** 2010-06-26

**Authors:** Marietjie Schutte, Hendrik G Visser, Andreas Roodt

**Affiliations:** a Department of Chemistry, University of the Free State, PO Box 339, Bloemfontein, 9300, South Africa

## Abstract

In the title salt, (C_8_H_20_N)[ReBr(C_7_H_5_O_2_)(CO)_3_], the Re^I^ atom is octa­hedrally surrounded by three facially orientated carbonyl ligands, one bidendate tropolonate ligand and a bromide ligand. The small O—Re—O bite angle of 74.88 (12)° leads to a distortion of the octa­hedral coordination sphere. The bromide ligand and the axial carbonyl ligand are substitutionally disordered over two positions in a 0.922 (3):0.078 (3) ratio. An array of C—H⋯O and C—H⋯Br hydrogen-bonding inter­actions between the cations and neighbouring rhenate anions stabilizes the crystal packing.

## Related literature

For the synthesis of the Re^I^-tricarbonyl synthon, see: Alberto *et al.* (1996 ▶). A range of related rhenium bidentate complexes have been characterized by Schutte & Visser (2008 ▶); Alberto *et al.* (1992 ▶, 1996 ▶, 1998 ▶); Abram *et al.* (1996 ▶); Findeisen & Schmidt (1991 ▶); Egli *et al.* (1997 ▶), Brasey *et al.* (2004 ▶); Gibson *et al.* (1999 ▶); Bochkova *et al.* (1987 ▶); Cheng *et al.* (1988 ▶); Mundwiler *et al.* (2004 ▶). For similar structures, see: Schutte *et al.* (2007 ▶, 2008 ▶) and for comparable Re—Br distances, see: Schutte *et al.* (2007 ▶, 2009 ▶).
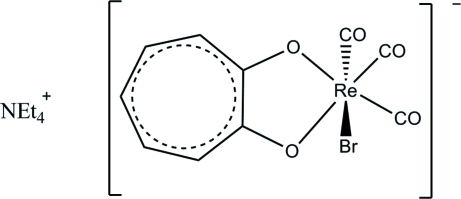


## Experimental

### 

#### Crystal data

(C_8_H_20_N)[ReBr(C_7_H_5_O_2_)(CO)_3_]
*M* *_r_* = 601.5
Monoclinic, 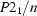

*a* = 12.334 (5) Å
*b* = 10.754 (5) Å
*c* = 16.053 (5) Å
β = 101.983 (5)°
*V* = 2082.9 (14) Å^3^
*Z* = 4
Mo *K*α radiation
μ = 7.78 mm^−1^
*T* = 100 K
0.58 × 0.18 × 0.17 mm

#### Data collection

Bruker SMART CCD diffractometer
Absorption correction: multi-scan (*SADABS*; Bruker, 2004 ▶) *T*_min_ = 0.196, *T*_max_ = 0.273
16808 measured reflections
5160 independent reflections
4700 reflections with *I* > 2σ(*I*)
*R*_int_ = 0.047

#### Refinement

*R*[*F* ^2^ > 2σ(*F* ^2^)] = 0.034
*wR*(*F* ^2^) = 0.086
*S* = 1.13
5160 reflections
251 parameters
3 restraints
H-atom parameters constrained
Δρ_max_ = 3.16 e Å^−3^
Δρ_min_ = −1.41 e Å^−3^

### 

Data collection: *SMART* (Bruker, 2005 ▶); cell refinement: *SAINT-Plus* (Bruker, 2004 ▶); data reduction: *SAINT-Plus*; program(s) used to solve structure: *SHELXS97* (Sheldrick, 2008 ▶); program(s) used to refine structure: *SHELXL97* (Sheldrick, 2008 ▶); molecular graphics: *DIAMOND* (Brandenburg & Putz, 2005 ▶); software used to prepare material for publication: *WinGX* (Farrugia, 1999 ▶).

## Supplementary Material

Crystal structure: contains datablocks global, I. DOI: 10.1107/S1600536810024505/wm2362sup1.cif

Structure factors: contains datablocks I. DOI: 10.1107/S1600536810024505/wm2362Isup2.hkl

Additional supplementary materials:  crystallographic information; 3D view; checkCIF report

## Figures and Tables

**Table 1:** Selected bond lengths (Å)

C1—Re1	1.906 (5)
C2—Re1	1.903 (5)
O11—Re1	2.126 (3)
O12—Re1	2.135 (3)
Re1—C3*A*	1.861 (7)
Re1—C3*B*	1.923 (18)
Re1—Br1*B*	2.467 (16)
Re1—Br1*A*	2.6334 (9)

**Table 2:** Hydrogen-bond geometry (Å, °)

*D*—H⋯*A*	*D*—H	H⋯*A*	*D*⋯*A*	*D*—H⋯*A*
C13—H13⋯O3*A*^i^	0.93	2.43	3.344 (7)	169
C25—H25*A*⋯Br1*A*^ii^	0.97	2.89	3.809 (5)	158
C26—H26*C*⋯O11	0.96	2.58	3.542 (7)	176
C27—H27*B*⋯O11	0.97	2.57	3.401 (6)	143

Symmetry codes: (i) 
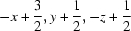
; (ii) 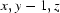.

**Fig. 1.**
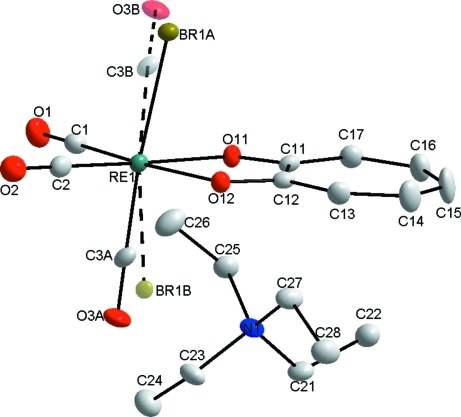
Representation of the molecular structure of the title compound, showing the numbering scheme and displacement ellipsoids drawn at the 50% probability level. Hydrogen atoms are omitted for clarity.

**Fig. 2.**
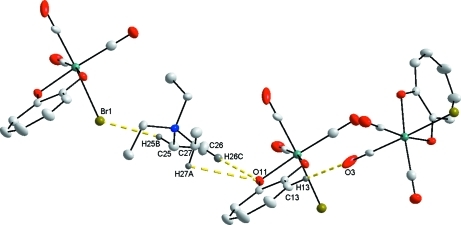
Representation of the hydrogen-bonding interactions and the packing of the title structure (only one orientation of the disordered Br/CO groups is shown).

## supplementary crystallographic information

### Comment

The title compound forms part of an ongoing study involving various bidentate ligands on the rhenium tricarbonyl core and the effects thereof, crystallographically as well as kinetically (Schutte & Visser, 2008; Schutte *et al.*, 2009). Various of these bidentate complexes have been synthesized before (Alberto *et al.*, 1992, 1996, 1998; Abram *et al.*, 1996; Findeisen & Schmidt, 1991; Egli *et al.*, 1997; Brasey *et al.*, 2004; Gibson *et al.*, 1999; Bochkova *et al.*, 1987; Cheng *et al.*, 1988; Mundwiler *et al.*, 2004). However, only a few *O,O*' bidentate ligands are known in literature.

The octahedral geometry around the Re^I^ metal centre is slightly distorted (Fig. 1) due to the effect of the small bite angle of 74.9 (1) °. Good correlations regarding bond distances and angles are found with related structures (Schutte *et al.*, 2007, 2008). The Re—Br bond distance of 2.6334 (9) Å compares well with 2.6270 (3) Å (Schutte *et al.*, 2007). The Re—O(bidentate) distances of 2.137 (3) Å and 2.125 (4) Å are well within the range of 2.123 (4) Å to 2.146 (4) Å observed for similar structures (Schutte *et al.*, 2007, 2008). Also the bite angles of 74.1 (2)° and 73.6 (7) °, (Schutte *et al.*, 2007, 2008) compare well with 74.88 (12)° found in the title structure.

An array of C—H···O and C—H···Br hydrogen-bonding interactions between rhenate anions and neighbouring cations stabilizes the crystal packing (Fig. 2).

### Experimental

0.04 mmol tropolone was dissolved in 3 ml of methanol and heated to 323 K. 0.039 mmol [NEt_4_]_2_[Re(CO)_3_Br_3_] (synthesized according to Alberto *et al.* (1996)) were added to the solution and left to stir overnight. The reaction mixture was left to stand and crystals suitable for single-crystal X-ray crystallography formed. The crystals were orange cuboids with a maximum edge length of about 0.6 mm.

### Refinement

The aromatic H atoms were placed in geometrically idealized positions and constrained to ride on their parent atoms with *U*_iso_(H) = 1.2*U*_eq_(C). The aliphatic H atoms were place in geometrically idealized positions and constrained to ride on their parent atoms with *U*_iso_(H) = 1.2*U*_eq_(C) for methylene carbon atoms and *U*_iso_(H) = 1.5*U*_eq_(C) for methyl atoms. Substitutional disorder between the bromide ligand and and the trans carbonyl ligand was observed in a 0.922 (3):0.078 (3) ratio. Such a kind of disorder has been observed in similar complexes and in Rh-Vaska compounds. The highest peak and the deepest hole in the final difference map are located 0.86 Å and 0.65 Å from Re1.

### Crystal data

**Table:**

(C_8_H_20_N)[ReBr(C_7_H_5_O_2_)(CO)_3_]	*F*(000) = 1160
*M**_r_* = 601.5	*D*_x_ = 1.918 Mg m^−^^3^
Monoclinic, *P*2_1_/*n*	Mo *K*α radiation, λ = 0.71073 Å
Hall symbol: -P 2yn	Cell parameters from 9467 reflections
*a* = 12.334 (5) Å	θ = 2.3–28.3°
*b* = 10.754 (5) Å	µ = 7.78 mm^−^^1^
*c* = 16.053 (5) Å	*T* = 100 K
β = 101.983 (5)°	Cuboid, orange
*V* = 2082.9 (14) Å^3^	0.58 × 0.18 × 0.17 mm
*Z* = 4	

### Data collection

**Table:**

Bruker SMART CCD diffractometer	4700 reflections with *I* > 2σ(*I*)
graphite	*R*_int_ = 0.047
phi and ω scans	θ_max_ = 28.3°, θ_min_ = 1.9°
Absorption correction: multi-scan (*SADABS*; Bruker, 2004)	*h* = −16→16
*T*_min_ = 0.196, *T*_max_ = 0.273	*k* = −14→12
16808 measured reflections	*l* = −21→21
5160 independent reflections	

### Refinement

**Table:**

Refinement on *F*^2^	Primary atom site location: structure-invariant direct methods
Least-squares matrix: full	Secondary atom site location: difference Fourier map
*R*[*F*^2^ > 2σ(*F*^2^)] = 0.034	Hydrogen site location: inferred from neighbouring sites
*wR*(*F*^2^) = 0.086	H-atom parameters constrained
*S* = 1.13	*w* = 1/[σ^2^(*F*_o_^2^) + (0.0337*P*)^2^ + 6.0941*P*] where *P* = (*F*_o_^2^ + 2*F*_c_^2^)/3
5160 reflections	(Δ/σ)_max_ = 0.002
251 parameters	Δρ_max_ = 3.16 e Å^−^^3^
3 restraints	Δρ_min_ = −1.41 e Å^−^^3^

### Special details

**Table:**

Geometry. All s.u.'s (except the s.u. in the dihedral angle between two l.s. planes) are estimated using the full covariance matrix. The cell s.u.'s are taken into account individually in the estimation of s.u.'s in distances, angles and torsion angles; correlations between s.u.'s in cell parameters are only used when they are defined by crystal symmetry. An approximate (isotropic) treatment of cell s.u.'s is used for estimating s.u.'s involving l.s. planes.
Refinement. Refinement of *F*^2^ against ALL reflections. The weighted *R*-factor *wR* and goodness of fit *S* are based on *F*^2^, conventional *R*-factors *R* are based on *F*, with *F* set to zero for negative *F*^2^. The threshold expression of *F*^2^ > 2σ(*F*^2^) is used only for calculating *R*-factors(gt) *etc*. and is not relevant to the choice of reflections for refinement. *R*-factors based on *F*^2^ are statistically about twice as large as those based on *F*, and *R*- factors based on ALL data will be even larger.

### Fractional atomic coordinates and isotropic or equivalent isotropic displacement parameters (Å^2^)

**Table:**

	*x*	*y*	*z*	*U*_iso_*/*U*_eq_	Occ. (<1)
C1	0.8439 (4)	0.5302 (5)	0.5663 (3)	0.0181 (9)	
C2	0.9102 (4)	0.7076 (5)	0.4752 (3)	0.0199 (10)	
C11	0.5498 (4)	0.5523 (4)	0.3763 (3)	0.0148 (9)	
C12	0.5849 (4)	0.6462 (4)	0.3215 (3)	0.0149 (9)	
C13	0.5209 (4)	0.6930 (5)	0.2455 (3)	0.0197 (10)	
H13	0.5552	0.7546	0.2196	0.024*	
C14	0.4145 (4)	0.6621 (6)	0.2027 (3)	0.0274 (12)	
H14	0.3896	0.7047	0.152	0.033*	
C15	0.3397 (4)	0.5777 (6)	0.2239 (3)	0.0310 (13)	
H15	0.2724	0.5697	0.1854	0.037*	
C16	0.3543 (4)	0.5036 (6)	0.2965 (3)	0.0247 (11)	
H16	0.2949	0.4524	0.3006	0.03*	
C17	0.4455 (4)	0.4958 (5)	0.3636 (3)	0.0205 (10)	
H17	0.4355	0.444	0.4077	0.025*	
C21	0.5919 (4)	0.0054 (5)	0.3394 (3)	0.0208 (10)	
H21A	0.6032	−0.0001	0.2814	0.025*	
H21B	0.6263	−0.0671	0.3698	0.025*	
C22	0.4687 (4)	0.0006 (6)	0.3370 (3)	0.0253 (11)	
H22A	0.4331	0.0706	0.3056	0.038*	
H22B	0.4563	0.003	0.394	0.038*	
H22C	0.4385	−0.075	0.3098	0.038*	
C23	0.7703 (4)	0.1180 (5)	0.3677 (3)	0.0212 (10)	
H23A	0.7689	0.1153	0.3071	0.025*	
H23B	0.8066	0.1947	0.3898	0.025*	
C24	0.8381 (5)	0.0097 (6)	0.4099 (4)	0.0290 (12)	
H24A	0.8041	−0.0668	0.3872	0.044*	
H24B	0.8416	0.0125	0.4702	0.044*	
H24C	0.9117	0.0146	0.3991	0.044*	
C25	0.6461 (4)	0.1209 (5)	0.4744 (3)	0.0208 (10)	
H25A	0.673	0.0413	0.4987	0.025*	
H25B	0.5693	0.1285	0.479	0.025*	
C26	0.7123 (5)	0.2234 (6)	0.5273 (3)	0.0303 (12)	
H26A	0.789	0.2158	0.5247	0.045*	
H26B	0.7045	0.2161	0.5854	0.045*	
H26C	0.685	0.303	0.5052	0.045*	
C27	0.5946 (4)	0.2388 (5)	0.3406 (3)	0.0218 (10)	
H27A	0.5218	0.2442	0.3544	0.026*	
H27B	0.6373	0.3097	0.3664	0.026*	
C28	0.5810 (5)	0.2483 (5)	0.2445 (3)	0.0261 (11)	
H28A	0.5453	0.3254	0.2251	0.039*	
H28B	0.5366	0.1802	0.2178	0.039*	
H28C	0.6525	0.2452	0.2298	0.039*	
N1	0.6509 (3)	0.1205 (4)	0.3806 (3)	0.0175 (8)	
O1	0.8829 (3)	0.4790 (4)	0.6285 (2)	0.0295 (9)	
O2	0.9905 (3)	0.7658 (4)	0.4826 (2)	0.0278 (8)	
O11	0.6230 (3)	0.5185 (3)	0.44163 (19)	0.0156 (6)	
O12	0.6850 (3)	0.6885 (3)	0.34718 (19)	0.0158 (6)	
Re1	0.778478 (14)	0.611175 (17)	0.462429 (10)	0.01392 (7)	
Br1A	0.66965 (4)	0.77746 (5)	0.53345 (3)	0.01591 (18)	0.922 (3)
C3A	0.8454 (5)	0.4916 (7)	0.4061 (4)	0.0190 (12)	0.922 (3)
O3A	0.8876 (5)	0.4139 (5)	0.3694 (3)	0.0257 (10)	0.922 (3)
Br1B	0.8340 (11)	0.4525 (12)	0.3685 (8)	0.043 (3)	0.078 (3)
C3B	0.718 (5)	0.741 (4)	0.522 (3)	0.0190 (12)	0.078 (3)
O3B	0.679 (5)	0.816 (4)	0.554 (4)	0.0257 (10)	0.078 (3)

### Atomic displacement parameters (Å^2^)

**Table:**

	*U*^11^	*U*^22^	*U*^33^	*U*^12^	*U*^13^	*U*^23^
C1	0.019 (2)	0.020 (3)	0.017 (2)	0.0019 (18)	0.0063 (16)	0.0018 (17)
C2	0.022 (2)	0.022 (3)	0.015 (2)	0.002 (2)	0.0019 (17)	0.0011 (17)
C11	0.019 (2)	0.015 (2)	0.0106 (18)	0.0009 (18)	0.0040 (15)	−0.0007 (16)
C12	0.019 (2)	0.012 (2)	0.0143 (19)	0.0004 (18)	0.0061 (16)	−0.0004 (16)
C13	0.020 (2)	0.025 (3)	0.015 (2)	−0.0010 (19)	0.0069 (17)	0.0039 (17)
C14	0.021 (3)	0.040 (3)	0.019 (2)	0.000 (2)	0.0005 (18)	0.013 (2)
C15	0.014 (2)	0.050 (4)	0.026 (3)	−0.003 (2)	−0.0043 (19)	0.013 (2)
C16	0.016 (2)	0.035 (3)	0.023 (2)	−0.003 (2)	0.0035 (18)	0.004 (2)
C17	0.021 (2)	0.025 (3)	0.017 (2)	−0.002 (2)	0.0064 (17)	0.0021 (19)
C21	0.027 (2)	0.016 (2)	0.021 (2)	−0.001 (2)	0.0083 (18)	−0.0033 (18)
C22	0.024 (3)	0.029 (3)	0.024 (2)	−0.003 (2)	0.0067 (19)	−0.005 (2)
C23	0.019 (2)	0.019 (3)	0.029 (2)	0.0013 (19)	0.0124 (19)	0.0008 (19)
C24	0.026 (3)	0.027 (3)	0.034 (3)	0.005 (2)	0.006 (2)	0.002 (2)
C25	0.024 (3)	0.022 (3)	0.018 (2)	0.000 (2)	0.0079 (18)	0.0031 (18)
C26	0.044 (3)	0.024 (3)	0.021 (2)	−0.005 (2)	0.004 (2)	−0.001 (2)
C27	0.029 (3)	0.018 (3)	0.020 (2)	0.004 (2)	0.0085 (18)	0.0035 (18)
C28	0.032 (3)	0.026 (3)	0.022 (2)	0.005 (2)	0.009 (2)	0.005 (2)
N1	0.020 (2)	0.014 (2)	0.0201 (19)	0.0030 (16)	0.0089 (15)	0.0022 (15)
O1	0.0248 (19)	0.042 (3)	0.0206 (17)	0.0058 (17)	0.0027 (14)	0.0102 (16)
O2	0.0224 (19)	0.029 (2)	0.0307 (19)	−0.0054 (16)	0.0031 (14)	0.0015 (16)
O11	0.0141 (15)	0.0185 (18)	0.0134 (14)	−0.0016 (13)	0.0009 (11)	0.0031 (12)
O12	0.0149 (15)	0.0179 (18)	0.0149 (14)	−0.0020 (13)	0.0036 (11)	0.0022 (12)
Re1	0.01479 (11)	0.01596 (11)	0.01091 (9)	−0.00006 (7)	0.00243 (6)	0.00038 (6)
Br1A	0.0174 (3)	0.0161 (3)	0.0149 (3)	0.0001 (2)	0.00479 (18)	−0.00174 (17)
C3A	0.022 (3)	0.021 (3)	0.015 (3)	−0.006 (2)	0.004 (2)	−0.005 (2)
O3A	0.025 (3)	0.025 (3)	0.029 (2)	0.008 (2)	0.012 (2)	−0.0041 (18)
Br1B	0.048 (7)	0.039 (7)	0.045 (6)	−0.014 (5)	0.017 (6)	0.010 (5)
C3B	0.022 (3)	0.021 (3)	0.015 (3)	−0.006 (2)	0.004 (2)	−0.005 (2)
O3B	0.025 (3)	0.025 (3)	0.029 (2)	0.008 (2)	0.012 (2)	−0.0041 (18)

### Geometric parameters (Å, °)

**Table:**

C1—O1	1.153 (6)	C23—H23A	0.97
C1—Re1	1.906 (5)	C23—H23B	0.97
C2—O2	1.156 (6)	C24—H24A	0.96
C2—Re1	1.903 (5)	C24—H24B	0.96
C11—O11	1.286 (5)	C24—H24C	0.96
C11—C17	1.398 (7)	C25—N1	1.520 (6)
C11—C12	1.462 (6)	C25—C26	1.521 (7)
C12—O12	1.300 (5)	C25—H25A	0.97
C12—C13	1.402 (6)	C25—H25B	0.97
C13—C14	1.389 (7)	C26—H26A	0.96
C13—H13	0.93	C26—H26B	0.96
C14—C15	1.385 (8)	C26—H26C	0.96
C14—H14	0.93	C27—C28	1.520 (6)
C15—C16	1.392 (7)	C27—N1	1.525 (6)
C15—H15	0.93	C27—H27A	0.97
C16—C17	1.390 (7)	C27—H27B	0.97
C16—H16	0.93	C28—H28A	0.96
C17—H17	0.93	C28—H28B	0.96
C21—C22	1.513 (7)	C28—H28C	0.96
C21—N1	1.517 (6)	O11—Re1	2.126 (3)
C21—H21A	0.97	O12—Re1	2.135 (3)
C21—H21B	0.97	Re1—C3A	1.861 (7)
C22—H22A	0.96	Re1—C3B	1.923 (18)
C22—H22B	0.96	Re1—Br1B	2.467 (16)
C22—H22C	0.96	Re1—Br1A	2.6334 (9)
C23—C24	1.510 (7)	C3A—O3A	1.201 (9)
C23—N1	1.529 (6)	C3B—O3B	1.123 (18)
			
O1—C1—Re1	178.7 (5)	H26A—C26—H26B	109.5
O2—C2—Re1	179.5 (5)	C25—C26—H26C	109.5
O11—C11—C17	117.7 (4)	H26A—C26—H26C	109.5
O11—C11—C12	116.1 (4)	H26B—C26—H26C	109.5
C17—C11—C12	126.2 (4)	C28—C27—N1	115.3 (4)
O12—C12—C13	118.4 (4)	C28—C27—H27A	108.4
O12—C12—C11	115.6 (4)	N1—C27—H27A	108.4
C13—C12—C11	126.0 (4)	C28—C27—H27B	108.4
C14—C13—C12	130.4 (5)	N1—C27—H27B	108.4
C14—C13—H13	114.8	H27A—C27—H27B	107.5
C12—C13—H13	114.8	C27—C28—H28A	109.5
C15—C14—C13	130.1 (5)	C27—C28—H28B	109.5
C15—C14—H14	114.9	H28A—C28—H28B	109.5
C13—C14—H14	114.9	C27—C28—H28C	109.5
C14—C15—C16	127.1 (5)	H28A—C28—H28C	109.5
C14—C15—H15	116.5	H28B—C28—H28C	109.5
C16—C15—H15	116.5	C21—N1—C25	108.7 (4)
C17—C16—C15	128.8 (5)	C21—N1—C27	111.2 (4)
C17—C16—H16	115.6	C25—N1—C27	107.9 (4)
C15—C16—H16	115.6	C21—N1—C23	108.3 (4)
C16—C17—C11	131.3 (5)	C25—N1—C23	111.8 (4)
C16—C17—H17	114.4	C27—N1—C23	109.0 (4)
C11—C17—H17	114.4	C11—O11—Re1	116.9 (3)
C22—C21—N1	115.3 (4)	C12—O12—Re1	116.4 (3)
C22—C21—H21A	108.4	C3A—Re1—C2	88.5 (2)
N1—C21—H21A	108.4	C3A—Re1—C1	87.7 (2)
C22—C21—H21B	108.4	C2—Re1—C1	87.6 (2)
N1—C21—H21B	108.4	C3A—Re1—C3B	176 (2)
H21A—C21—H21B	107.5	C2—Re1—C3B	88 (2)
C21—C22—H22A	109.5	C1—Re1—C3B	92.1 (19)
C21—C22—H22B	109.5	C3A—Re1—O11	94.4 (2)
H22A—C22—H22B	109.5	C2—Re1—O11	174.40 (17)
C21—C22—H22C	109.5	C1—Re1—O11	97.33 (17)
H22A—C22—H22C	109.5	C3B—Re1—O11	89 (2)
H22B—C22—H22C	109.5	C3A—Re1—O12	93.6 (2)
C24—C23—N1	114.4 (4)	C2—Re1—O12	100.19 (16)
C24—C23—H23A	108.7	C1—Re1—O12	172.18 (16)
N1—C23—H23A	108.7	C3B—Re1—O12	87.1 (18)
C24—C23—H23B	108.7	O11—Re1—O12	74.88 (12)
N1—C23—H23B	108.7	C3A—Re1—Br1B	10.7 (4)
H23A—C23—H23B	107.6	C2—Re1—Br1B	96.0 (3)
C23—C24—H24A	109.5	C1—Re1—Br1B	95.6 (3)
C23—C24—H24B	109.5	C3B—Re1—Br1B	171.5 (19)
H24A—C24—H24B	109.5	O11—Re1—Br1B	86.3 (3)
C23—C24—H24C	109.5	O12—Re1—Br1B	84.8 (3)
H24A—C24—H24C	109.5	C3A—Re1—Br1A	175.61 (19)
H24B—C24—H24C	109.5	C2—Re1—Br1A	94.80 (15)
N1—C25—C26	115.4 (4)	C1—Re1—Br1A	95.34 (14)
N1—C25—H25A	108.4	C3B—Re1—Br1A	7.5 (19)
C26—C25—H25A	108.4	O11—Re1—Br1A	82.07 (9)
N1—C25—H25B	108.4	O12—Re1—Br1A	83.01 (9)
C26—C25—H25B	108.4	Br1B—Re1—Br1A	165.0 (3)
H25A—C25—H25B	107.5	O3A—C3A—Re1	179.3 (6)
C25—C26—H26A	109.5	O3B—C3B—Re1	177 (6)
C25—C26—H26B	109.5		

### Hydrogen-bond geometry (Å, °)

**Table:**

*D*—H···*A*	*D*—H	H···*A*	*D*···*A*	*D*—H···*A*
C13—H13···O3A^i^	0.93	2.43	3.344 (7)	169
C25—H25A···Br1A^ii^	0.97	2.89	3.809 (5)	158
C26—H26C···O11	0.96	2.58	3.542 (7)	176
C27—H27B···O11	0.97	2.57	3.401 (6)	143

Symmetry codes: (i) −*x*+3/2, *y*+1/2, −*z*+1/2; (ii) *x*, *y*−1, *z*.
